# Identification of Sortilin Alternatively Spliced Variants in Mouse 3T3L1 Adipocytes

**DOI:** 10.3390/ijms22030983

**Published:** 2021-01-20

**Authors:** Ashley Lui, Robert Sparks, Rekha Patel, Niketa A. Patel

**Affiliations:** 1 Department of Molecular Medicine, Morsani College of Medicine, University of South Florida, Tampa, FL 33612, USA; ashleylui@usf.edu (A.L.); rpsparks@usf.edu (R.S.); 2 Research Service, James A. Haley Veteran’s Hospital, Tampa, FL 33612, USA; rekha.patel1@va.gov

**Keywords:** Sortilin, Glut4, alternative splicing, adipocytes, molecular dynamics, protein–protein docking

## Abstract

Type 2 diabetes mellitus is a metabolic disorder defined by systemic insulin resistance. Insulin resistance in adipocytes, an important regulator of glucose metabolism, results in impaired glucose uptake. The trafficking protein, sortilin, regulates major glucose transporter 4 (Glut4) movement, thereby promoting glucose uptake in adipocytes. Here, we demonstrate the presence of an alternatively spliced sortilin variant (Sort^17b^), whose levels increase with insulin resistance in mouse 3T3L1 adipocytes. Using a splicing minigene, we show that inclusion of alternative exon 17b results in the expression of Sort^17b^ splice variant. Bioinformatic analysis indicated a novel intrinsic disorder region (IDR) encoded by exon 17b of Sort^17b^. Root mean square deviation (RMSD) and root mean square fluctuation (RMSF) measurements using molecular dynamics demonstrated increased flexibility of the protein backbone within the IDR. Using protein–protein docking and co-immunoprecipitation assays, we show robust binding of Glut4 to Sort^17b^. Further, results demonstrate that over-expression of Sort^17b^ correlates with reduced Glut4 translocation and decreased glucose uptake in adipocytes. The study demonstrates that insulin resistance in 3T3L1 adipocytes promotes expression of a novel sortilin splice variant with thus far unknown implications in glucose metabolism. This knowledge may be used to develop therapeutics targeting sortilin variants in the management of type 2 diabetes and metabolic syndrome.

## 1. Introduction

Type 2 Diabetes Mellitus (T2DM), affecting more than 400 million people worldwide, is a chronic progressive metabolic disorder with no cure. In recent decades, the prevalence of T2DM has been increasing to affect nearly 10% of the population, while the age of onset of T2DM has been decreasing [1]. Systemic insulin resistance is a hallmark of T2DM resulting in endocrine dysfunction and increased glucose levels in circulation. The resultant hyperglycemia causes systemic inflammation and promotes comorbidities such as heart disease, fatty liver disease, and neurodegeneration.

The correlation between adiposity and T2DM is intricately studied, revealing an independent increased risk for T2DM in overweight and obese patients [2]. Adipocytes are critical energy regulators of glucose homeostasis. Circulating glucose is taken up by adipocytes and retained as energy stores. In adipocytes, the major glucose transporter, glucose transporter-4 (Glut4), is held in specialized storage vesicles sized 50 to 70 nm [3], also known as Glut4 storage vesicles (GSVs). Basally, GSVs are tethered in cytoplasmic holding sites, GSVs translocate to the plasma membrane with insulin stimulation and later recycle back through the trans-golgi network (TGN) [4,5]. Insulin resistant adipocytes demonstrate aberrant GSV trafficking. This reduction in Glut4 movement to the plasma membrane ultimately results in excess glucose in circulation. Several proteins are central to Glut4 trafficking, translocation and degradation of GSV. Mouse 3T3L1 pre-adipocytes are a well-established model for adipogenesis and insulin sensitive glucose uptake, and are predominantly utilized for evaluating Glut4 trafficking. Studies using 3T3L1 adipocytes identified major components of GSVs including SNARES [6], Rab GTPases [7], insulin-regulated aminopeptidase (IRAP) [8], and sortilin [9]. Many of these studies showed varied functional components of GSVs present at different stages of trafficking; however, sortilin has been shown to be the major protein in GSVs [10]. Furthermore, sortilin is “essential and sufficient” for GSV formation. Sortilin knockdown in 3T3L1 pre-adipocytes leads to the loss of GSV development that is normally stimulated with adipocyte maturation [11].

Alternative splicing is a common and significant post-transcriptional method of gene regulation used to generate different proteins with distinct functions from a single gene. More than 90% of human genes are alternatively spliced. Aberrant alternative splicing can cause disease or affect disease susceptibility or severity [12]. This critical control over gene expression occurs in response to environmental factors and is often developmental, disease, or tissue specific. Alternative splicing is indicated in several studies on glucose metabolism [13,14] and diabetes [15,16]. Alternatively spliced variants of sortilin are previously described in frontotemporal lobar degeneration of the brain [17]. Expression of alternatively spliced variants of sortilin in adipocytes has not been demonstrated yet.

Sortilin is a single pass trans-membrane protein consisting of a large luminal domain including a multi-ligand binding VPS10p domain whose 10 conserved cysteines (10CC domain) are crucial for ligand interaction [18,19]. Sortilin’s N-terminal domain binds the first luminal loop of Glut4 aiding in localization and response to signaling [20]. Sortilin has a brief 50 residue motif-rich cytosolic C-terminal tail. The C-terminal domain motifs enable sortilin to form complexes to regulate trafficking within the TGN. Mutations of specific sites of sortilin’s cytoplasmic C-terminal domain impair retromer protein-directed trafficking from the plasma membrane to the cytoplasm and inhibit linkage of GSVs to the plasma membrane via GGA clathrin adaptors [21]. Such studies emphasize sortilin’s role in insulin resistance and GSV cellular localization.

Since insulin resistance in T2DM decreases glucose uptake and sortilin is a major component of GSVs, we evaluated sortilin in 3T3L1 adipocytes under conditions rendering it resistant to insulin action. Our results showed presence of an alternatively spliced variant of sortilin, which was not yet described in the literature, in 3T3L1 adipocytes. Hence, we evaluated the role of this novel alternatively spliced sortilin variant due to sortilin’s pivotal role in maintaining glucose homeostasis in adipocytes. 

## 2. Results

### 2.1. Sortilin Alternatively Spliced Variant Expression during Adipogenesis

**Figure 1:**
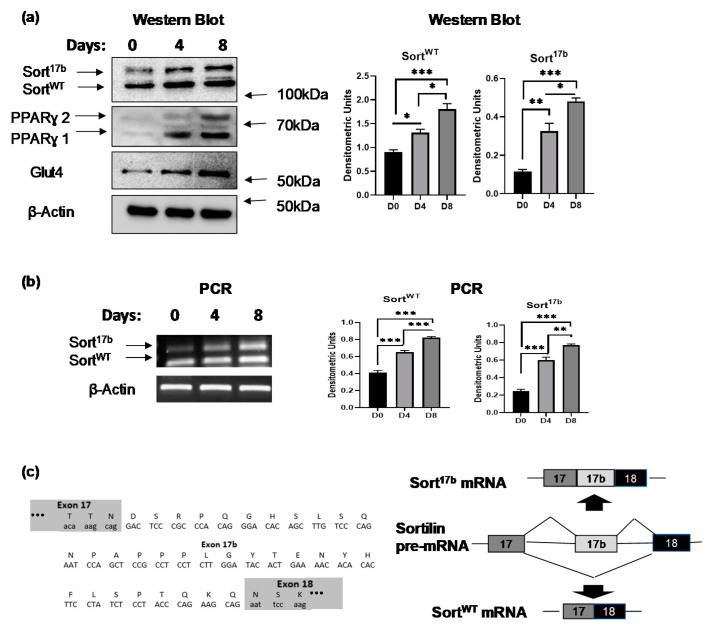
Sortilin alternatively spliced variants’ expression during adipogenesis. 3T3L1 preadipocytes were differentiated and harvested at days 0 (pre-adipocytes), day 4 (terminally differentiated adipocytes), and day 8 (mature adipocytes). (**a**) Western blot analysis was performed on lysates harvested on days 0, 4 and 8 and probed using antibodies against sortilin, peroxisome proliferator activated receptor ɣ (PPARγ), glucose transporter-4 (Glut4) or β-actin as indicated in the figure. Graphs show densitometric analysis of individual bands for each protein that are normalized to β-actin (*n* = 5). Statistical analysis was performed by one-way ANOVA; * *p* < 0.05, ** *p* < 0.01, and *** *p* < 0.001 between day 0 and day 4 samples or day 0 and day 8 samples. (**b**) RNA was isolated on days 0, 4 and 8, followed by PCR analysis using primers specific to sortilin exon 17 (sense) to exon 19 (anti-sense). The PCR products were separated on a 1% agarose gel and visualized using ethidium bromide staining. Graphs show densitometric analysis of individual PCR products normalized to β-actin (*n* = 5). Statistical analysis was performed by one-way ANOVA; ** *p* < 0.01, and *** *p* < 0.001 between days 0, 4, and 8 samples for wildtype (Sort^WT^) and sortilin variant with included exon 17b (Sort^17b^) variants. (**c**) The two PCR products obtained using primers to sortilin exon 17 (sense) to exon 19 (anti-sense) were purified and sequenced. Sequencing results revealed inclusion of a 99 bp sequence, named exon 17b, between exons 17 and 18 of sortilin mRNA. The schematic depicts sortilin pre-mRNA alternative splicing generating Sort^WT^ mRNA (exclusion of exon 17b) and Sort^17b^ mRNA (inclusion of exon 17b). Sortilin alternatively spliced variants’ expression during adipogenesis. 3T3L1 preadipocytes were differentiated and harvested at days 0 (pre-adipocytes), day 4 (terminally differentiated adipocytes), and day 8 (mature adipocytes). (**a**) Western blot analysis was performed on lysates harvested on days 0, 4 and 8 and probed using antibodies against sortilin, peroxisome proliferator activated receptor ɣ (PPARγ), glucose transporter-4 (Glut4) or β-actin as indicated in the figure. Graphs show densitometric analysis of individual bands for each protein that are normalized to β-actin (*n* = 5). Statistical analysis was performed by one-way ANOVA; * *p* < 0.05, ** *p* < 0.01, and *** *p* < 0.001 between day 0 and day 4 samples or day 0 and day 8 samples. (**b**) RNA was isolated on days 0, 4 and 8, followed by PCR analysis using primers specific to sortilin exon 17 (sense) to exon 19 (anti-sense). The PCR products were separated on a 1% agarose gel and visualized using ethidium bromide staining. Graphs show densitometric analysis of individual PCR products normalized to β-actin (*n* = 5). Statistical analysis was performed by one-way ANOVA; ** *p* < 0.01, and *** *p* < 0.001 between days 0, 4, and 8 samples for wildtype (Sort^WT^) and sortilin variant with included exon 17b (Sort^17b^) variants. (**c**) The two PCR products obtained using primers to sortilin exon 17 (sense) to exon 19 (anti-sense) were purified and sequenced. Sequencing results revealed inclusion of a 99 bp sequence, named exon 17b, between exons 17 and 18 of sortilin mRNA. The schematic depicts sortilin pre-mRNA alternative splicing generating Sort^WT^ mRNA (exclusion of exon 17b) and Sort^17b^ mRNA (inclusion of exon 17b).

*In vitro* differentiation of 3T3L1 pre-adipocytes to mature adipocytes results in the expression of multiple proteins including those necessary to respond to insulin and promote glucose uptake. 3T3L1 adipocytes were differentiated over a course of 8 days and harvested on day 0 (pre-adipocyte), day 4 (terminally differentiated adipocyte), and day 8 (mature adipocyte). Western blot analysis was performed using antibodies against sortilin N-terminal domain showing two bands with approximately 5 kDa difference. Peroxisome proliferator activated receptor ɣ (PPARɣ) expression served as a marker of adipogenesis (Figure 1a). Interestingly, the expression levels of the higher band of sortilin increased from day 4 onwards when the cells were terminally differentiated.

Two alternatively spliced variants of sortilin were previously described in murine neurons [17,22]: wildtype (Sort^WT^) and sortilin variant with included exon 17b (Sort^17b^). To evaluate whether the two protein bands observed in Figure 1a were generated via alternative splicing of sortilin pre-mRNA, total RNA was extracted from differentiating 3T3L1 cells on days 0, 4, and 8. To simultaneously evaluate expression of both spliced variants, PCR was performed using primers for sortilin exon 17 (sense) and exon 19 (antisense) and products were separated on a 1% agarose gel (Figure 1b). Both PCR sortilin products were purified from agarose gel and the sequencing results confirmed a 99bp insertion between exon 17 and exon 18 of sortilin mRNA (Figure 1c).

### 2.2. Sortilin Expression in Other Adipose Depots and Tissue

**Figure 2:**
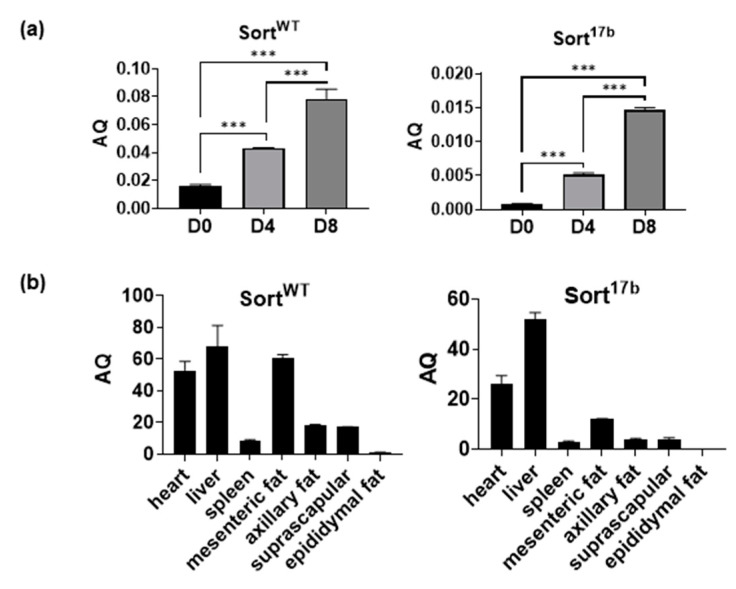
Endogenous expression levels of murine sortilin variants. (**a**) Total RNA was isolated from differentiating 3T3L1 cells harvested at days 0, 4, and 8. Real-time SYBR Green qPCR was performed using primers specifically targeting the 17/18 exon junction (to detect Sort^WT^) or within exon 17b (to detect Sort^17b^). A standard curve for each sortilin variant was included with the qPCR assay along with samples run in triplicate. Absolute quantification (AQ, ng) for Sort^WT^ and Sort17b was calculated by normalizing the values to β-actin (*n* = 5). Statistical analysis was performed by one-way ANOVA; *** *p* < 0.001 between day 0, 4 and 8 samples for Sort^WT^ and Sort^17b^ variants. (**b**) Organs and specific adipose depots were harvested from C57BL6 mice and homogenized in a bead homogenizer. RNA was isolated and real-time SYBR Green qPCR was performed using primers specifically targeting the 17/18 exon junction (to detect Sort^WT^) or within exon 17b (to detect Sort^17b^). Absolute quantification (AQ, ng) for Sort^WT^ and Sort17b was calculated by normalizing the values to β-actin (*n* = 4).

Next, results were verified using SYBR Green Real Time qPCR with primers specifically targeting the 17/18 exon junction (to detect Sort^WT^) or within exon 17b (to detect Sort^17b^). Absolute quantification of mRNA was achieved by including a standard curve. Results showed that Sort^WT^ was the predominant sortilin spliced variant with concurrent detection of lower levels of Sort^17b^ (Figure 2a). The overall expression of sortilin increased with adipogenesis, which concurred with the data obtained for protein levels. This is, to our knowledge, the first demonstration of alternative splicing of sortilin during the differentiation of adipocytes. Since day 4 showed distinct expression of both sortilin variants, future experiments were predominantly conducted using differentiated adipocytes at day 4, unless otherwise stated.

To determine the presence and levels of sortilin variants in other adipose depots and organs, C57BL6 mice were euthanized and tissues were harvested. RNA was isolated and SYBR Green Real Time qPCR was performed (Figure 2b) to determine absolute levels of sortilin variants using the primers described above for Figure 2a. Sort^WT^ and Sort^17b^ levels were high in heart and liver, and within adipose depots both sortilin variants were most abundant in mesenteric adipose tissue.

### 2.3. Induction of Insulin Resistance by High Serum Promotes Sortilin Exon 17b Inclusion

**Figure 3:**
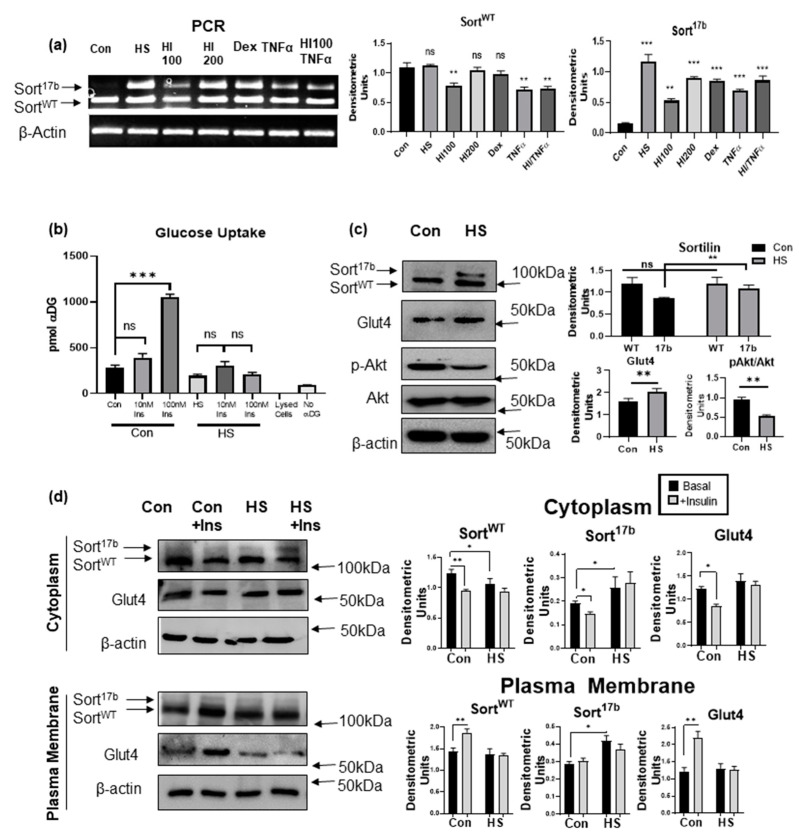
Insulin resistance induced in 3T3L1 adipocytes results in increased Sort^17b^. (**a**) 3T3L1 pre-adipocytes (day 0) were differentiated (as described in methods) for 2 days. Insulin resistance methods were then induced (10% high serum (HS), 100 nM insulin (HI100), 200 nM insulin (HI200), 1µM dexamethasone (Dex), 2.5 nM TNFα, and 100 nM insulin + 2.5 nM TNFα) for 48 h. RNA was isolated and PCR was performed using primers specific to sortilin exon 17 (sense) and exon 19 (anti-sense) and run on a 1% agarose gel Bands were visualized using ethidium bromide staining and normalized to β-actin expression. (*n* = 5). Graph represents densitometric units normalized to β-actin for each variant and represents five experiments performed independently. Statistical analysis was performed by one-way ANOVA; ** *p* < 0.01, and *** *p* < 0.001 between no treatment and each treated sample. Ns = not significant. (**b**) 3T3L1 pre-adipocytes were differentiated and on day 4, adipocytes were treated with 10% high serum (HS) for 48 h. Insulin stimulated glucose uptake assay was performed and uptake of α-deoxy glucose (αDG) was measured; no αDG and lysed cells were used as controls (*n* = 5). Statistical analysis was performed by one-way ANOVA; *** *p* < 0.001 between no treatment and 10 nM or 100 nM insulin treated cells in control and high serum (HS) samples. Ns = not significant. (**c**) 3T3L1 pre-adipocytes were differentiated and on day 4, adipocytes were treated with 10% high serum (HS) for 48 h. Whole cell lysates were harvested and western blot analysis was performed using antibodies against sortilin, Glut4, pAkt, Akt or β-actin as indicated in the figure. Graphs show densitometric analysis of individual bands for each protein that are normalized to β-actin. Statistical analysis was performed by two tailed *t*-test; ** *p* < 0.01 between control and high serum (HS) samples. Ns = not significant. (*n* = 5). (**d**) 3T3L1 pre-adipocytes were differentiated and on day 4, adipocytes were treated with 10% high serum (HS) for 48 h. Prior to fractionation, 100 nM insulin was added for 10 min. Adipocytes were fractionated to yield a cytoplasmic protein fraction and plasma membrane protein fraction. Western blot analysis was performed using antibodies against sortilin, Glut4 or β-actin as indicated in the figure (*n* = 4). Graphs show densitometric analysis of individual bands for each protein that are normalized to β-actin. Statistical analysis was performed by one-way ANOVA; * *p* < 0.05, and ** *p* < 0.01 between control and high serum (HS) samples and basal vs. insulin stimulated samples.

To evaluate if sortilin alternatively spliced variants relate to impaired glucose uptake and insulin resistance as seen in the pathology of T2DM, 3T3L1 adipocytes were rendered insulin resistant using several previously established methods. 3T3L1 preadipocytes on day 0 have low levels of Sort^17b^ which increase with differentiation. To enable detection of both sortilin variants’ levels in response to methods rendering them insulin resistant, 3T3L1 pre-adipocytes (day 0) were differentiated (as described in methods) for 2 days. In separate wells, along with the differentiation cocktail, several methods to induce insulin resistance were undertaken including: 10% high serum (HS), 100 nM and 200 nM hyperinsulinemia (HI100, HI200), 1 μM dexamethasone (Dex), 2.5 nM TNFα, and a combination of hyperinsulinemia and TNFα. After 48 h, RNA was isolated followed by PCR analysis using primers for exon 17 (sense) and exon 19 (antisense) which detects Sort^WT^ and Sort^17b^ expression simultaneously. Results (Figure 3a) demonstrate that all methods of insulin resistance promoted an increase in inclusion of sortilin exon 17b, whereas the high serum method consistently and reproducibly increased expression of Sort^17b^. The high serum method was previously used to induce insulin resistance in adipocytes [23,24]. Hence, we next verified that the high serum method mimicked the physiological response of insulin resistance by performing a glucose uptake assay. 3T3L1 pre-adipocytes were differentiated and on day 4, high (10%) serum was added for 48 h. Glucose uptake assay was then performed using Promega’s Glo glucose uptake assay kit. Stimulation with 100 nM insulin induced a dramatic increase in glucose uptake in control adipocytes, but glucose uptake was significantly impaired in high serum treated cells (Figure 3b), indicating that this method induced insulin resistance in 3T3L1 adipocytes. 

Next, we evaluated the protein expression levels of sortilin’s alternatively spliced variants under conditions of high serum-induced insulin resistance in 3T3L1 adipocytes. 3T3L1 pre-adipocytes were differentiated and, on day 4, high (10%) serum was added for 48 h. Whole cell lysate was harvested for western blot analysis and results showed an increase in Sort^17b^ protein in high serum treated adipocytes compared to control (Figure 3c). Consistent with insulin resistance, phosphorylation of AKT at site Ser473 was also markedly reduced. Interestingly, levels of Glut4 were also increased in high serum treated cells, suggesting decreased glucose uptake to be independent of total Glut4 levels.

Basally Glut4 protein is tethered within the cytoplasm of adipocytes in Glut4 storage vesicles (GSVs). Under insulin sensitive conditions, insulin signaling cascades stimulate GSV translocation to the plasma membrane for glucose uptake. 3T3L1 adipocytes, on day 4, were rendered insulin resistant in high serum for 48 h and then treated with 100 nM insulin (10 min) followed by fractionation of cytoplasm and plasma membrane. Results showed a marked reduction in Glut4 and sortilin translocation to the plasma membrane from cytosolic fractions compared to control (Figure 3d) in high serum samples. This reduction in insulin stimulated Glut4 translocation is indicative of adipocyte dysfunction and insulin resistance, as noted in T2DM. 

### 2.4. Induction of Insulin Resistance by High Serum Promotes Sortilin Exon 17b Inclusion

**Figure 4:**
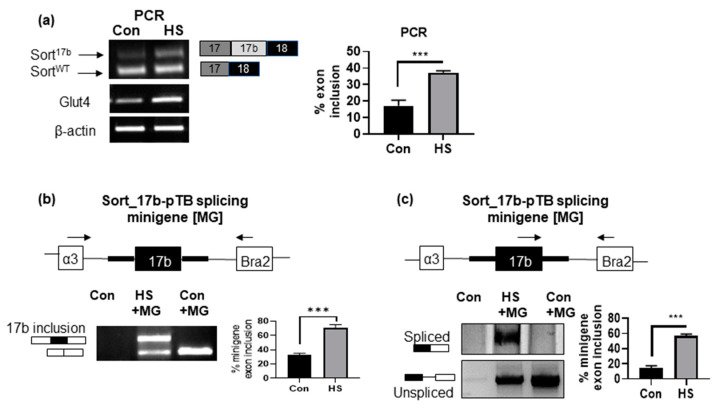
High serum (HS) method of insulin resistance promotes inclusion of sortilin exon 17b. (**a**) 3T3L1 cells were differentiated and on day 4, insulin resistance was induced by high serum for 48 h. Total RNA was isolated, PCR was performed using primers specific to sortilin exon 17 (sense) to exon 19 (anti-sense) to simultaneously detect both sortilin variants. The PCR products were separated on a 1% agarose gel, visualized using ethidium bromide staining and normalized to β-actin expression. (*n* = 5). Graph represents endogenous percent inclusion of exon 17b, which was calculated using the Equation (1). Statistical analysis was performed by two tailed *t*-test; *** *p* < 0.001 between control and high serum (HS) samples. *(***b**) Sort_17b-pTB splicing minigene (MG) was transfected into 3T3L1 adipocytes and treated with high serum for 48 h. The control samples had no minigene transfection. Total RNA was isolated, PCR was performed using primers on splice donor exon (α3, sense) to splice acceptor exon (Bra2, antisense) of the Sort_17b-pTB splicing minigene. The PCR products were separated on a 1% agarose gel and visualized using ethidium bromide staining (*n* = 5). Graph represents percent exon 17b inclusion within Sort_17b-pTB splicing minigene (MG) in control vs. high serum, which was calculated using the Equation (2). Statistical analysis was performed by two tailed *t*-test; *** *p* < 0.001 between control and high serum (HS) samples. (**c**) PCR was performed using primer specific for sortilin exon 17b (sense) and splice acceptor exon (Bra2, antisense). The PCR products were separated on PAGE and visualized using silver staining (*n* = 5). Graph represents percent exon 17b inclusion within Sort_17b-pTB splicing minigene (MG) in control vs. high serum, which was calculated using the Equation (2). Statistical analysis was performed by two tailed *t*-test; *** *p* < 0.001 between control and high serum (HS) samples.

We evaluated if insulin resistant conditions induced by high serum promotes inclusion of exon 17b. 3T3L1 pre-adipocytes were differentiated and on day 4, adipocytes were treated with 10% high serum (HS) for 48 h. Total RNA was isolated and PCR was performed using primers that detect both variants simultaneously (as described above). Results demonstrate a significant increase in sortilin exon 17b inclusion, resulting in increased levels of Sort^17b^ mRNA (Figure 4a) concurrent with an increase in Glut4 mRNA expression. These results agree with protein levels observed above in Figure 3c.

(1) $$\%exon\;inclusion=\;\frac{\mathrm{Sort}\;17b}{\mathrm{Sort}\;17b+\mathrm{Sort}\;\mathrm{WT}}\times 100.$$

(2) $$\%exon\;inclusion=\;\frac{17b\;spliced\;product}{17b\;spliced\;product\;+unspliced\;vector}\times 100$$

We next sought to validate that the increased expression of Sort^17b^ in high serum-induced insulin resistance in 3T3L1 adipocytes was due to alternative splicing, a post-transcriptional event. To do so, we utilized a sortilin minigene in which sortilin exon 17b with flanking introns was cloned into pTB splicing vector (a kind gift from Dr. Leonard Petrucelli [17]). The Sort_17b-pTB splicing minigene was transfected overnight and then cells were treated with high serum for 48 h. RNA was harvested and PCR performed using primers specific to splice donor exon (α3, sense primer) and to splice acceptor exon (Bra2, antisense primer) as well as primers specific to exon 17b to splice acceptor exon (Figure 4b,c). Results show increased exon 17b inclusion in the splicing minigene assay in response to high serum compared to control. These results demonstrate that high serum treatment promotes expression of Sort^17b^ via alternative splicing.

### 2.5. Inclusion of Sortilin Exon 17b Introduces an Intrinsically Disordered Region in Sort^17b^ Protein

**Figure 5:**
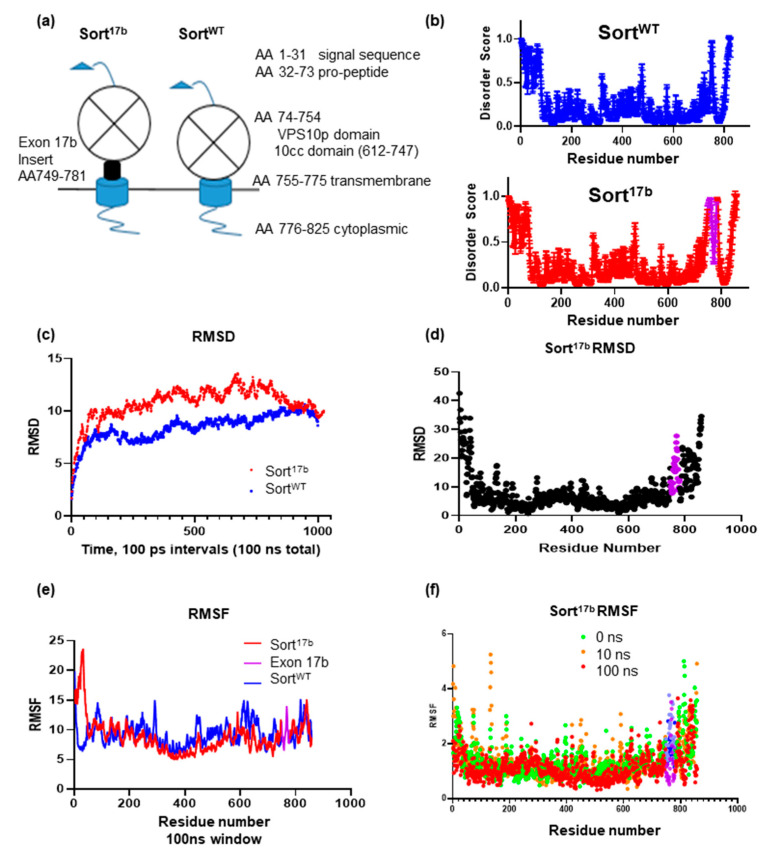
Inclusion of exon 17b in Sort^17b^ variant results in an intrinsically disordered region. (**a**) Schematic of sortilin depicting its amino acid (AA) domain consisting of a signal sequence and pro-peptide that is cleaved early during processing. The luminal domain includes the large VPS10p domain and 10CC domain. There is a single brief helical transmembrane and a short cytoplasmic domain. Exon 17b is inserted immediately prior to the transmembrane domain on the luminal side of the sortilin protein. (**b**) Sortilin protein sequence was analyzed using the disorder predictor PONDR-Fit (Predictor of Naturally Disordered Regions), which calculates an average disorder score and standard error score per residue. Amino acids 748 to 780 represent sortilin exon 17b marked in purple. Any scores above 0.5 are considered disordered. (**c**) Full length Sort^17b^ and Sort^WT^ were prepared with ITASSER, minimized using Schrodinger software, and molecular dynamics (MD) was run for 100 ns using NAMD2.12 and CHARMM36m force field. (**d**) Root mean square deviation (RMSD) per residue was calculated using Visual Molecular Dynamics (VMD), indicating a broader deviation amongst residues included in the 33 amino acid insertion of exon 17b (purple). (**e**) Root mean square fluctuation (RMSF) per residue was calculated using VMD timeline over a 100 ns window, Sort^WT^ C-terminal residues were shifted 33 amino acids to align with Sort^17b^ sequence and exon 17b is marked in purple. (**f**) RMSF per residue for Sort^17b^ was calculated using VMD timeline of 0 (green), 10 (orange) and 100 ns (red) using a 5 ns window indicating fluctuation amongst residues contained in the 33 amino acid insertion (purple).

Sortilin is a large protein consisting of a multiligand binding VPS10p domain at the amino terminus leading into a single pass transmembrane domain and a short cytosolic tail. The insertion of exon 17b is between the 10cc motif of the VPS10p domain and the transmembrane domain (Schematic in Figure 5a). Since the amino acid sequence specifies the 3D structure of protein, which in turn affects its function, we evaluated whether the sortilin exon 17b insertion introduced any intrinsically disordered regions (IDRs) within the otherwise ordered domains [25]. Intrinsic disorder is a measure of lack of protein folding resulting in increased solvent exposure and flexibility for binding partners, which may affect receptor-ligand affinity of proteins [26]. The widely used computational tool and meta-predictor PONDR-Fit (Predictor of Natural Disordered Regions) was used to analyze the Sort^WT^ and Sort^17b^ protein sequences. PONDR-Fit calculates an average disorder score and standard error per residue and thus predicts disorder [25,27]. Results (Figure 5b) demonstrate that the inclusion of amino acids 748 to 780 represents sortilin exon 17b (marked in purple) producing an IDR.

Since there are no full-length structures of sortilin from cryo-EM in its native membrane bound state, we used molecular modeling to visualize both sortilin variants. Mouse Sort^WT^ extracellular domain was determined by X-Ray crystallography (Protein Database (PDB) ID: 5NMR); however, the structures of the transmembrane and cytoplasmic tail domains have not been determined. Using amino acid sequences of both sortilin variants, we obtained structural predictions from the protein server iTASSER [27,28,29], an online server for determining lowest energy folding compositions for *de novo* protein predictions utilizing available structural information of similar homologues of a given protein or portions of proteins. Sortilin structural prediction results were aligned with the X-Ray crystallography generated PDB structure of the sortilin VPS10p domain. Then, proteins were equilibrated for 100 ns using molecular dynamics (MD) run with NAMD 2.12 [30] using the CHARMM36m force field [31]. MD simulations were run to observe protein structural changes in sortilin including backbone flexibility (using root mean square deviation (RMSD)) [32,33] and molecular motions (using root mean square fluctuation (RMSF)). During equilibration, RMSD was plotted over a 100 ns time trajectory (Figure 5c). Sort^17b^ showed significantly higher RMSD than Sort^WT^ over the first 75 ns of the simulation, suggesting increased backbone flexibility. To further probe which residues may contribute to increased flexibility, RMSD was plotted per residue for Sort^17b^ (Figure 5d), indicating movement within exon 17b and the C-terminal domain of sortilin.

RMSF aids in identifying short sequences or residues contributing to structural flexibility that may affect binding of sortilin to ligands. Plotting the RMSF over the course of the 100 ns simulation (Figure 5e) showed most residues of sortilin to be fairly stable. To test whether the residues of exon 17b fluctuated over the course of the simulation, we sampled each residue at three time points: 0 ns (green), 10 ns (orange) and 100 ns (red) using a 5 ns window (Figure 5f). Interestingly, there was increased fluctuation of residues within exon 17b in the beginning compared to the end of the simulation. This further indicates that the inserted 33 amino acids of exon 17b contributes to flexibility of residues in Sort^17b^. When coupling Sort^17b^ RMSF with RMSD results, both backbone and residue movement demonstrates that the 33 amino acid insert introduces a disordered region. Notably within exon 17b, residues 767, 768, and 769, had similar beginning and end behavior showing a focused region within the exon insertion. This analysis sheds light on the relationship between alternative splicing, a post-transcriptional event to post-translational events that define function of the protein.

### 2.6. Sort^17b^ Splice Variant Is a Strong Binding Partner of Glut4

**Figure 6:**
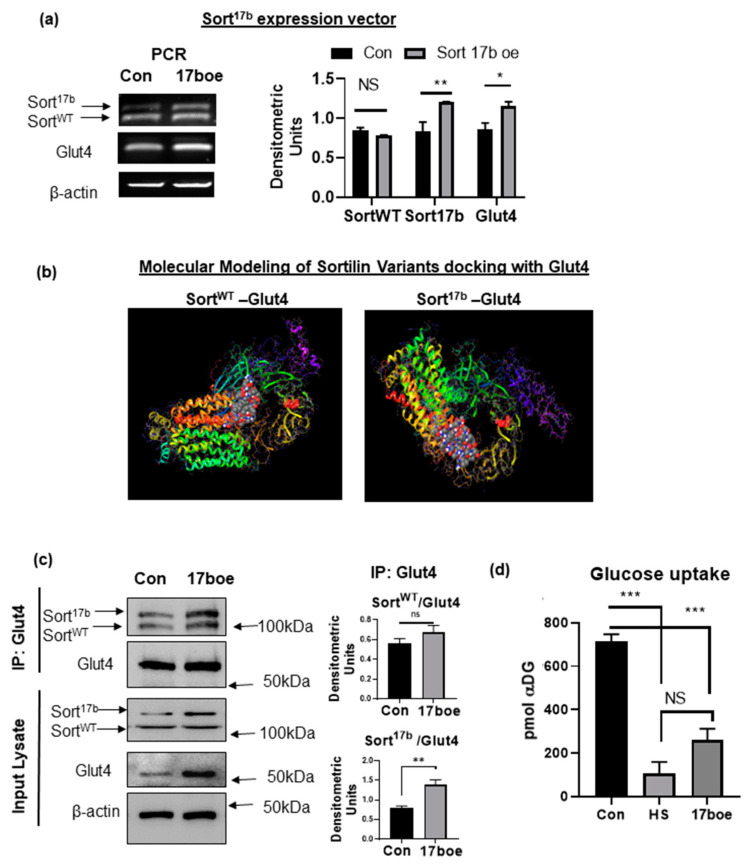
Sort^17b^ binds robustly to Glut4 (**a**) Sort^17b^ expression vector was transfected in 3T3L1 preadipocytes for 48 h (17boe sample) or day 2 control (Con sample). Total RNA was isolated, PCR was performed using primers specific to sortilin exon 17 (sense) to exon 19 (anti-sense) to simultaneously detect both sortilin variants. The PCR products were separated on a 1% agarose gel, visualized using ethidium bromide staining and normalized to β-actin expression (*n* = 5). Graph represents densitometric units representative of 5 experiments performed independently. Statistical analysis was performed by two tailed *t*-test; * *p* < 0.05, ** *p* < 0.01 between control and Sort^17b^ over-expressing (17b oe) samples. (**b**) Molecular dynamics was performed to elucidate binding of Sort^WT^ or Sort^17b^ to Glut4. Equilibrated and minimized Glut4, Sort^WT^ and Sort^17b^ proteins were docked to the equilibrated and minimized Glut4 protein. Docking results show binding of Sort^WT^ as well as Sort^17b^ to the first luminal loop of Glut4. Transmembrane helices of Glut4 are seen on the left, the first luminal domain of Glut4 is indicated by ball model, and sortilin’s luminal 10 blade propeller domain can be seen on the right with the C-terminal tail in purple. (**c**) Sort^17b^ expression vector was transfected in 3T3L1 preadipocytes for 48 h (17 boe sample) or day 2 control (Con sample). Coimmunoprecipitation assay was performed by using Glut4 antibody for immunoprecipitation. Western blot analysis was then performed on the immunoprecipitated (IP) samples using antibodies against sortilin or Glut4 as indicated in the figure. Graphs show densitometric analysis of individual sortilin bands normalized to Glut4 (*n* = 5). Simultaneously, the input lysate was analyzed by western blot using antibodies against sortilin or Glut4 (*n* = 5). Statistical analysis was performed by two tailed *t*-test; ** *p* < 0.01 between control and Sort^17b^ over-expressing (17 boe) samples. (**d**) 3T3L1 pre-adipocytes were differentiated and, on day 4, adipocytes were treated with high serum (HS) to induce insulin resistance or transfected with Sort^17b^ expression vector (17 boe sample) for 48 h. Insulin stimulated glucose uptake assay was performed and uptake of α- deoxy glucose (αDG) was measured; no αDG and lysed cells were used as controls (*n* = 5). Statistical analysis was performed by one-way ANOVA; *** *p* < 0.001 between no treatment and insulin treated cells in control and high serum (HS) samples or control and Sort17boe sample. Ns = not significant.

Sortilin is a central component of GSV and is pivotal in translocation of glucose transporter-4 (Glut-4) to facilitate glucose uptake. Since high serum-induced insulin resistance resulted in decreased glucose uptake and increase in expression of Sort^17b^ splice variant, we sought to evaluate the interaction of sortilin splice variants with Glut4 in 3T3L1 adipocytes. Sort^17b^ expression vector (gifted by Dr. Leonard Petrucelli [17]) was transfected into 3T3L1 adipocytes for 48 h. Over-expression of the Sort^17b^ was verified through PCR analysis (Figure 6a). Interestingly, Sort^17b^ over-expression increased Glut4 expression levels, similar to the increase seen under high serum conditions.

Exon 17b inclusion occurs within the 10cc domain directly above the transmembrane α helix of sortilin, an important region in sortilin ligand binding. Sortilin is a multiligand receptor and, in adipocytes, it is central for glucose metabolism by its interactions with Glut4 in GSVs and guiding proteins. Due to this insertion in a crucial ligand binding domain, we sought to determine if Glut4-sortilin interaction would be altered with Sort^17b^ compared to Sort^WT^. Using the equilibrated and minimized sortilin and Glut4 models described above, ClusPro server was used to dock each sortilin variant to the first luminal loop of Glut4, which is the domain where Glut4 binds to sortilin, as described in the literature [20] (Figure 6b). Results show Glut4 binding to luminal domain of Sort^17b^ as well as to Sort^WT.^ These results indicate that the insertion of exon 17b does not interfere with Glut4 binding to sortilin. 

To verify binding of Glut4 with Sort^17b^ splice variant, a co-immunoprecipitation assay was performed. Sort^17b^ was over-expressed in 3T3L1 adipocytes. Glut4 antibody was used to immunoprecipitate associated proteins in control and adipocytes over-expressing Sort^17b^. Results (Figure 6c) show that Glut4 binds to both Sort^WT^ and Sort^17b^. In Sort^17b^ over-expressing adipocytes, results show a significantly higher association of Sort^17b^ with Glut4.

In order to determine Sort^17b^’s role in insulin stimulated glucose uptake, Sort^17b^ plasmid was overexpressed in 3T3L1 adipocytes (as described above) followed by Promega’s Glo glucose uptake assay. 3T3L1 adipocytes were separately rendered insulin resistant using high serum and this sample was used as a control for the assay. Adipocytes overexpressing Sort^17b^ showed dramatically less uptake from basal levels compared to control cells in response to 100 nM insulin treatment (Figure 6d).

## 3. Discussion

Classically alternative splicing was shown to be tissue and development-specific. However, it is now established that alternative splicing is the norm and more than 95% of genes are alternatively spliced. Aberrant expression of splice variants is sometimes the cause of disease, while, in other scenarios, disease states alter expression of splice variants. Sortilin is a multiligand receptor and trafficking protein that is constitutively expressed in multiple organs. Alternatively, spliced sortilin variants were identified in the murine brain with an inserted exon between exons 17 and 18 in sortilin mRNA [17,22]. This alternatively spliced sortilin variant 17b in brain is shown to be increased in neuronal rodent models of front-temporal dementia where it plays a role in progranulin turnover. Of importance to note here is that the ligand for sortilin and its function is specific to the organ. Sortilin in the brain is described as a 110 kDa protein which was also observed in these 3T3L1 adipocytes; however, additional bands were also detected at approximately 60 and 64 kDa. Lower bands may be cleaved sortilin products and further research is needed to evaluate these products. We have observed (unpublished N. Patel lab data) increased exon 17b inclusion in sortilin pre-mRNA in human adipose tissue obtained from type 2 diabetic as well as from obese subjects compared to lean, normal subjects. Experiments are underway to analyze additional human samples such that a statistically significant cohort of age-matched male vs. female, normal vs. type 2 diabetic, lean vs. obese subjects can be analyzed at mRNA and protein levels and reported.

In adipose tissue, sortilin is a crucial trafficking protein in the TGN that also regulates major glucose transporter-4′s (Glut4) location in the cell. The majority of the GSV pool is cytoplasmic with low levels on the membrane, basally 5–10% in adipocytes [10]. Trafficking of Glut4 has proven to be complicated, but the role of sortilin is shown to be important in GSV movement in response to stimuli [9], post translational modifications, and ligand interactions [34]. Notably, post translational modifications of sortilin result in movement of sortilin vesicles to and from the lysosomal pathway [34,35,36,37], which may provide insight into Glut4 recycling in adipocytes.

Other methods were used in this study to induce insulin resistance in 3T3L1 adipocytes such as treatment with high insulin, dexamethasone, and TNFα. These methods also showed increased Sort^17b^ levels; however, the high serum method was steadily reproducible with significant increases in Sort^17b^ levels. To maintain uniformity throughout the experiments, we pursued this method for our evaluations. Serum is a rich mixture of proteins, hormones and growth factors which can affect gene expression and mimics the heterologous environment in vivo. We have initiated a separate project to identify a specific hormone or growth factor which may directly regulate expression of Sort^17b^. 

Our computational analysis data showed the introduction of an intrinsically disordered region with sortilin exon 17b inclusion. Intrinsic disorder in proteins increases exposure to solvent and increases potential interactions with other ligands and enzymes. The insertion of exon 17b exists at a crucial point in the sequence of sortilin, occurring before the single transmembrane segment and after the 10CC domain of the amino terminus, which is shown to affect ligand interaction [18,19]. Functional changes of intrinsic disorder are difficult to predict, but due to their dynamic nature we anticipated this insertion of 33 amino acids may alter physiological functions of sortilin. This was further explored through MD simulations indicating that Sort^17b^ includes residues that are highly flexible with subtle differences compared to Sort^WT^ structure over the course of 100 ns simulations. Thus, we tested if this insertion affects ligand binding using protein–protein docking of sortilin variants to Glut4. Our results indicated that the first luminal loop of Glut4, as previously described [20], bound the 10-bladed β-propellor structure of Sort^WT^ and Sort^17b^. Further, the data suggest that the Glut4 interaction site is distinct from the sites where sortilin VPS10p ligands, such as PCSK9 and neurotensin, canonically bind to sortilin [38,39]. 

To directly test sortilin variant interaction with known ligand Glut4 in adipocytes, we performed a co-immunoprecipitation assay. Surprisingly, we saw enhanced binding of Sort^17b^ to Glut4 with a dramatic decrease in glucose uptake. It is unclear how Sort^17b^ contributes to dysfunctional Glut4 trafficking (as indicated in fractionation assay) since it maintains its ability to bind ligands using its luminal domain and maintains cytosolic motifs for binding guiding proteins. This suggests altered structural implications of the inserted exon 17b affecting physiological characteristics in adipocytes. We plan to continue experiments using Sort^17b^ plasmids in the future to determine binding characteristics, cellular locations of sortilin and Glut4, and changes in post translational modifications.

Sortilin can be cleaved by proteases and released into the extracellular matrix [40,41] or secreted in exosomes [42]. It is possible that the intrinsic disordered region introduces new cleavage sites above the transmembrane domain of sortilin. The neuronal murine Sort^17b^ levels were associated with higher secreted and soluble sortilin in the conditioned media [17,43]. We have also detected increased secretion of sortilin with Sort^17b^ overexpression and in high serum induced insulin resistant 3T3L1 adipocytes. The functional implication of this finding in adipocytes is also under investigation. 

Here, we demonstrate the presence of alternatively spliced variants of sortilin in mouse 3T3L1 adipocytes and their differential expression in insulin resistant conditions, which underlie T2DM and metabolic syndrome. We elucidated the phenotype of Sort^17b^ in 3T3L1 adipocyte glucose homeostasis, indicating that Sort^17b^ plays a role in the pathogenesis of T2DM in mouse models. Understanding the role of sortilin’s alternatively spliced variants may bridge a critical gap in knowledge regarding glucose transporter mis-localization in insulin resistant mouse adipocytes and lead to novel targets for therapeutics in T2DM management. 

## 4. Materials and Methods

### 4.1. Cell Culture and Reagents

Mouse 3T3-L1 preadipocytes were purchased from ATCC^®^ CL-173™ and passaged as preconfluent cultures in Dulbecco’s Modification of Eagle’s Medium (DMEM) high glucose (Invitrogen, Carlsbad, CA, USA) with 10% newborn calf serum (Sigma-Aldrich, St. Louis, MO, USA) at 37 °C and 10% CO_2_. Once confluent (day 0), cells were differentiated in DMEM high glucose with 10% fetal bovine serum (Atlas Biological, Fort Collins, CO, USA), 10 μg/mL bovine insulin (Sigma), 1 mM dexamethasone (Sigma), and 0.5 mM isobutyl-1-methylxanthine (Sigma). On day 2, media was replaced with DMEM high glucose, 10% FBS, and bovine insulin. Day 4 and onwards, cells were cultured in DMEM high glucose plus 10% FBS. To induce insulin resistance, cells were treated for 48 h with 10% horse serum, 100 nM or 200 nM insulin, 5 nM TNFα, 1 μM dexamethasone, or combination of 100 nM Insulin and TNFα with media change. All cell culture media and reagents were purchased from Sigma. 

### 4.2. Polymerase Chain Reaction and qPCR- SYBR Green

Total RNA was isolated from cells using Trizol^TM^ (Thermo Fisher Scientific) as per the manufacturer’s instructions. A quantity of 1 µg of RNA (260/230 > 1.8 and 260/290 > 1.8) was used to synthesize cDNA using ReadyScript^TM^ synthesis mix (Sigma RDRT). In addition, 1 µL of cDNA was amplified using JumpStart REDTaq ReadyMix Reaction Mix (P0982); products were run on a 1% agarose gel and imaged in ProteinSimple FluorChem M^TM^. Densitometric analysis was performed using AlphaView Software. Primers used included Sortilin exon 17 S 5′-CAAATGCCAAGGTGGGATGAA-3′ and sortilin exon 19 AS 5′-TGACAAGCATCAGTCCCACGAT-3′ to see splice variants, sortilin exon 17b S 5′-AATCCAGCTCTGCCTCCTCT-3′, Glut4 S 5′-GGTGTGGTCAATACGGTCTTCAC-3′ and AS 5′-AGCAGAGCCACGGTCATCAAGA-3′, and β-actin S 5′ GTGGGCCGCTCTAGGCACCAA-3′ and AS 5′-CTCTTTGATGTCACGCACGATTTC-3′. For qPCR absolute quantification, amplification was performed on the Viia 7 (ABI) and primer concentrations were optimized to give a single melt curve. Plate set up included a standard series, no template control and no reserve transcriptase control. A standard curve was generated and used to calculate absolute quantities of target expression using primers above. Samples were normalized to β-actin for absolute quantification.

### 4.3. Animals

C57Bl6 mice (Harlan) were procured, raised, and studied in pathogen-free environments. Mice were housed in plastic, sawdust-covered cages with normal light–dark cycle and free access to chow and water. All protocols were reviewed and approved by the Institutional Animal Care and Use Committee at J. A. Haley Veteran’s Hospital and the University of South Florida, Division of Comparative Medicine (Protocol #4413V approved 02/03/2017). Nine-month-old male mice were euthanized under CO_2_ anesthesia and cervical dislocation was used as a secondary means of mortality. Tissues were rapidly harvested and immediate frozen in liquid nitrogen and stored in −80 °C until analyzed. Tissue was homogenized in Trizol^TM^ (Thermo Fisher Scientific) using a bead homogenizer, RNA was isolated and PCR was performed as described above.

### 4.4. Minigene Expression and Sortilin Plasmid Overexpression

Mouse sortilin plasmids were kindly gifted by Dr. Leonard Petrucelli (Mayo Clinic) and include a pTB mouse sortilin 17b minigene and pYX-ASC mouse sortilin 17b variant plasmid. Quantities of 2 μg per 35 mm plate were used for transfections (Lipofectamine 2000, as per manufacturer’s instructions) and cells were harvested after 48 h. Primers for the pTB minigene included α2-3 S 5′- CAACTTCAAGCTCCTAAGCCACTGC -3′ and Bra2 AS 5′- TAGGATCCGGTCACCAGGAAGTTGGTTAAATCA -3′.

### 4.5. Western Blot Analysis

Cell lysates (50 µg) were harvested using lysis buffer (Cell Signaling 9803S) + 10% protease/phosphatase inhibitor (Pierce A32957, A32953) then sonicated briefly. Samples were separated on a 7% SDS-PAGE gel. Proteins were electrophoretically transferred to nitrocellulose membranes and blocked with 5% nonfat dried milk in Tris-Buffered Saline with 0.05% Tween 20 (TBST). Membranes were probed with Sortilin (ab16640), Glut4 (Cell Signaling #2213), pAkt (Ser 473, Cell Signaling #4058), Akt2 (Cell Signaling #2962), and β-Actin (Sigma A3854). Secondary HRP antibodies were purchased from Biorad for rabbit (5196-2504) and mouse (0300-0108P). Incubation with chemiluminesence (Pierce 32109) was used for detection and images were digitally captured using ProteinSimple FluorChem M^TM^ and densitometric analysis was performed using AlphaView Software.

### 4.6. Co-Immunoprecipitation Assay

3T3-L1 adipocytes were harvested and 200 µL of 1 μg/μL of lysate was used for coimmunoprecipitation assay. Lysates were rocked with Protein A/G Agarose (Santa Cruz sc2003) for 30 min at 4 °C to clear nonspecific binding and centrifuged at 2000rpm for 1 min. The supernatant weas rocked with 2 μg Glut4-agarose antibody (Santa Cruz sc53566 AC) overnight at 4 °C and pellet was washed and resuspended in Laemmli buffer followed by western blot analysis as described above.

### 4.7. Glucose Uptake Assay

Glucose uptake was performed using ProMega Glo Assay (TM467) using a Luciferase reporter as per manufacturer’s instructions. Briefly, cells were serum starved in KRPH buffer for an hour, treated with 10 nM or 100 nM insulin for 10 min, and αdeoxyglucose (αDG) was added. Cells were washed and lysed. For αDG detection, luminescence was read on a Biotek Synergy Mx microplate reader using a 1 s integration time and αDG quantification of samples were calculated using an αDG standard curve.

### 4.8. Cell Fractionation

Cells were treated with 100 nM insulin for 10 min before harvesting in cold PBS and fractionated. In brief, cells were spun for 10 min at 1000 g and pellet suspended in buffer A (20 mM Tris, 250 mM sucrose, 1.2 mM EGTA, 2.5 mM MgCl_2_, 10% protease/phosphatase inhibitor). Lysate was sonicated for 10 s and spun at 105,000*g* for 30 min and resultant supernatant was collected as cytoplasmic fraction. Pellet was resuspended in buffer B (20 mM Tris, 250 mM sucrose, 5 mM EGTA, 2 mM EDTA, 1% Triton X, 10% protease inhibitor). Lysate was sonicated for 5 s and left on ice for 30 min before centrifugation at 105,000 g for 45 min. Supernatant was taken as membrane fraction and pellet discarded. Western blot was performed as described above. 

### 4.9. Disorder Analysis

Sortilin protein sequences were analyzed using PONDR-Fit (www.disprot.org), a multi-residue meta-predictor of intrinsic disorder in protein sequences. PONDR-Fit uses an eight-fold cross-validation method to calculate a disorder score and standard error per residue. Scores above 0.5 are considered disordered. 

### 4.10. Computational Modeling

Sequences for full length mouse Glut4 and Sort^wt^ were obtained from UniProt [44] and Sort^17B^ alternative splice variant sequences were derived via translation of Sort^17B^ nucleotide sequencing and alignment with Sort^wt^. Sort^WT^ and Sort^17b^ amino acid sequences were submitted to Iterative Threading Assembly Refinement (I-TASSER) protein folding server [27,28,29], where Sort^WT^ was aligned to mouse sortilin luminal domain (PDB ID: 5NMR). Mouse Glut4 protein (Swiss Model in Expasy (P14142)) and folded sortilin proteins were minimized in Schrodinger protein preparation wizard (2019-1: Maestro, Schrödinger, LLC, New York, NY, USA). Molecular dynamics simulations were done using NAMD 2.12 [30] and CHARMM36m force field [31] at a 100 ns time scale. The system was prepared using the CHARMM-GUI solution builder, with a salt concentration of 150 mM NaCl. Visualization and analysis was done using Visual Molecular Dynamics (VMD 1.9.3) [45]. The system was equilibrated for 10 ns restraining the Cα atoms of the protein (1.0 kcal/mol/A2) to allow for solvation. Root mean square deviations (RMSD) using backbone Cα atoms was calculated using VMD RMSD trajectory tool for Sort^WT^ and Sort^17b^. Root mean squared fluctuations (RMSF) of Cα atoms of Sort^17b^ were calculated using VMD timeline over the course of the whole simulation using both a 100 ns window and 5 ns windows for 3 time points (0, 10, and 100 ns), which were used to determine fluctuation of residues over the trajectory of the simulation. RMSD and RMSF results were exported and plotted using Graphpad Prism 8.4.3. Protein–protein docking was performed using the last frame from the equilibrated structures of Sort^17b^, Sort^WT^, and Glut4 on the ClusPro [46] server with poses chosen that indicated the first luminal loop of Glut4 binding to Sort^WT^ and Sort^17B^.

### 4.11. Statistical Analysis

All experiments were repeated 3–5 times as biological replicates and experimental samples run in triplicate to ensure reproducibility of results. Analyses were performed using PRISM^TM^ software and analyzed using two-tailed Student’s *t*-test, one-way or two-way ANOVA as indicated in figure legends. * *p* < 0.05, ** *p* < 0.01, and *** *p* < 0.001 were used as significant measures.

## Data Availability

All data is contained within this article and is available upon request.

## Abbreviations

T2DM	Type 2 Diabetes Mellitus
GSV	Glut4 storage vesicles
IDR	Intrinsic disordered region

## References

[1] Centers for Disease Control and Prevention (2017). National Diabetes Statistics Report, 2017.

[2] Hanley A.J.G., Wagenknecht L.E. (2008). Abdominal Adiposity and Diabetes Risk: The Importance of Precise Measures and Longitudinal Studies. Diabetes.

[3] Stöckli J., Fazakerley D.J., James D.E. (2011). GLUT4 exocytosis. J. Cell Sci..

[4] Bryant N.J., Govers R., James D.E. (2002). Regulated transport of the glucose transporter GLUT4. Nat. Rev. Mol. Cell Biol..

[5] Klip A., McGraw T.E., James D.E. (2019). 30 sweet years of GLUT4. J. Biol. Chem..

[6] Tamori Y., Kawanishi M., Niki T., Shinoda H., Araki S., Okazawa H., Kasuga M. (1998). Inhibition of Insulin-induced GLUT4 Translocation by Munc18c through Interaction with Syntaxin4 in 3T3-L1 Adipocytes. J. Biol. Chem..

[7] Reed S.E., Hodgson L.R., Song S., May M.T., Kelly E.E., McCaffrey M.W., Mastick C.C., Verkade P., Tavaré J. (2013). A role for Rab14 in the endocytic trafficking of GLUT4 in 3T3-L1 adipocytes. J. Cell Sci..

[8] Yeh T.-Y.J., Sbodio J.I., Tsun Z.-Y., Luo B., Chi N.-W. (2007). Insulin-stimulated Exocytosis of GLUT4 Is Enhanced by IRAP and Its Partner Tankyrase. Biochem. J..

[9] Blondeau N., Béraud-Dufour S., Lebrun P., Hivelin C., Coppola T. (2019). Sortilin in Glucose Homeostasis: From Accessory Protein to Key Player?. Front. Pharmacol..

[10] Morris N.J., Ross S.A., Lane W.S., Moestrup S.K., Petersen C.M., Keller S.R., Lienhard G.E. (1998). Sortilin Is the Major 110-kDa Protein in GLUT4 Vesicles From Adipocytes. J. Biol. Chem..

[11] Shi J., Kandror K.V. (2005). Sortilin is essential and sufficient for the formation of Glut4 storage vesicles in 3T3-L1 adipocytes. Dev. Cell.

[12] Ward A.J., Cooper T.A. (2010). The pathobiology of splicing. J. Pathol..

[13] Marcheva B., Perelis M., Weidemann B.J., Taguchi A., Lin H., Omura C., Kobayashi Y., Newman M.V., Wyatt E.J., McNally E.M. (2020). A role for alternative splicing in circadian control of exocytosis and glucose homeostasis. Genes Dev..

[14] Kleiman E., Carter G., Ghansah T., Patel1 N.A., Cooper D.R. (2009). Developmentally spliced PKCβII provides a possible link between mTORC2 and Akt kinase to regulate 3T3-L1 adipocyte insulin-stimulated glucose transport. Biochem. Biophys. Res. Commun..

[15] Dlamini Z., Mokoena F., Hull R. (2017). Abnormalities in alternative splicing in diabetes: Therapeutic targets. J. Mol. Endocrinol..

[16] Doumatey A.P., Xu H., Huang H., Trivedi N.S., Lei L., Elkahloun A., Adeyemo A., Rotimi C.N. (2015). Global Gene Expression Profiling in Omental Adipose Tissue of Morbidly Obese Diabetic African Americans. J. Endocrinol. Metab..

[17] Prudencio M., Jansen-West K.R., Lee W.C., Gendron T.F., Zhang Y.-J., Xu Y.-F., Gass J., Stuani C., Stetler C., Rademakers R. (2012). Misregulation of human sortilin splicing leads to the generation of a nonfunctional progranulin receptor. Proc. Natl. Acad. Sci. USA.

[18] Quistgaard E.M., Groftehauge M.K., Madsen P., Pallesen L.T., Christensen B., Sorensen E.S., Nissen P., Petersen C.M., Thirup S.S. (2014). Revisiting the structure of the Vps10 domain of human sortilin and its interaction with neurotensin. Protein Sci..

[19] Westergaard U.B., Sørensen E.S., Hermey G., Nielsen M.S., Nykjær A., Kirkegaard K., Jacobsen C., Gliemann J., Madsen P., Petersen C.M. (2004). Functional Organization of the Sortilin Vps10p Domain. J. Biol. Chem..

[20] Chernoff J. (2012). The first luminal loop confers insulin responsiveness to glucose transporter 4. Mol. Biol. Cell.

[21] Nielsen M.S., Madsen P., Christensen E.I., Nykjaer A., Gliemann J., Kasper D., Pohlmann R., Petersen C.M. (2001). The sortilin cytoplasmic tail conveys Golgi-endosome transport and binds the VHS domain of the GGA2 sorting protein. EMBO J..

[22] Polymenidou M., Lagier-Tourenne C., Hutt K.R., Huelga S.C., Moran J., Liang T.Y., Ling S.-C., Sun E., Wancewicz E., Mazur C. (2011). Long pre-mRNA depletion and RNA missplicing contribute to neuronal vulnerability from loss of TDP-43. Nat. Neurosci..

[23] Chamberlain J.M., O’Dell C., Sparks C.E., Sparks J.D. (2013). Insulin suppression of apolipoprotein B in McArdle RH7777 cells involves increased sortilin 1 interaction and lysosomal targeting. Biochem. Biophys. Res. Commun..

[24] Sparks J.D., Magra A.L., Chamberlain J.M., O’Dell C., Sparks C.E. (2016). Insulin dependent apolipoprotein B degradation and phosphatidylinositide 3-kinase activation with microsomal translocation are restored in McArdle RH7777 cells following serum deprivation. Biochem. Biophys. Res. Commun..

[25] Xue B., Dunbrack R.L., Williams R.W., Dunker A.K., Uversky V.N. (2010). PONDR-FIT: A meta-predictor of intrinsically disordered amino acids. Biochim. Biophys. Acta.

[26] Mollica L., Bessa L.M., Hanoulle X., Jensen M.R., Blackledge M., Schneider R. (2016). Binding Mechanisms of Intrinsically Disordered Proteins: Theory, Simulation, and Experiment. Front. Mol. Biosci..

[27] Roy A., Kucukural A., Zhang Y. (2010). I-TASSER: A unified platform for automated protein structure and function prediction. Nat. Protoc..

[28] Yang J., Yan R., Roy A., Xu D., Poisson J., Zhang Y. (2015). The I-TASSER Suite: Protein structure and function prediction. Nat. Methods.

[29] Yang J., Zhang Y. (2015). I-TASSER server: New development for protein structure and function predictions. Nucleic Acids Res..

[30] Phillips J.C., Braun R., Wang W., Gumbart J., Tajkhorshid E., Villa E., Chipot C., Skeel R.D., Kale L., Schulten K. (2005). Scalable molecular dynamics with NAMD. J. Comput. Chem..

[31] Huang J., Rauscher S., Nawrocki G., Ran T., Feig M., de Groot B.L., Grubmuller H., MacKerell A.D. (2017). CHARMM36m: An improved force field for folded and intrinsically disordered proteins. Nat. Methods.

[32] Maiorov V.N., Crippen G.M. (1994). Significance of Root-Mean-Square Deviation in Comparing Three-dimensional Structures of Globular Proteins. J. Mol. Biol..

[33] Monzon A.M., Zea D.J., Fornasari M.S., Saldaño T.E., Fernandez-Alberti S., Tosatto SC E., Parisi G. (2017). Conformational diversity analysis reveals three functional mechanisms in proteins. PLoS Comput. Biol..

[34] Sun S., Yang J., Xie W., Peng T., Lv Y. (2020). Complicated trafficking behaviors involved in paradoxical regulation of sortilin in lipid metabolism. J. Cell Physiol..

[35] McCormick P.J., Dumaresq-Doiron K., Pluviose A.-S., Pichette V., Tosato G., Lefrancois S. (2008). Palmitoylation Controls Recycling in Lysosomal Sorting and Trafficking. Traffic.

[36] Pan X., Zaarur N., Singh M., Morin P., Kandror K. (2017). VSortilin and retromer mediate retrograde transport of Glut4 in 3T3-L1 adipocytes. Mol. Biol. Cell.

[37] Dumaresq-Doiron K., Jules F., Lefrancois S. (2013). Sortilin turnover is mediated by ubiquitination. Biochem. Biophys. Res. Commun..

[38] Sparks R.P., Arango A.S., Aboff Z.L., Jenkins J.L., Guida W.C., Tajkhorshid E., Sparks C.E., Sparks J.D., Fratti R.A. (2019). Non-Canonical Binding of a Small Molecule to Sortilin Alters Cellular Trafficking of ApoB and PCSK9 in Liver Derived Cells. bioRxiv.

[39] Trabjerg E., Abu-Asad N., Wan Z., Kartberg F., Christensen S., Rand K.D. (2019). Investigating the conformational response of the Sortilin receptor upon binding endogenous peptide- and protein ligands by HDX-MS. Structure.

[40] Hermey G., Sjøgaard S.S., Petersen C.M., Nykjær A., Gliemann J. (2006). Tumour necrosis factor α-converting enzyme mediates ectodomain shedding of Vps10p-domain receptor family members. Biochem. J..

[41] Navarro V., Vincent J.-P., Mazella J. (2002). Shedding of the luminal domain of the neurotensin receptor-3/sortilin in the HT29 cell line. Biochem. Biophys. Res. Commun..

[42] Wilson C.M., Naves T., Vincent F., Melloni B., Bonnaud F., Lalloué F., Jauberteau M.-O. (2014). Sortilin mediates the release and transfer of exosomes in concert with two tyrosine kinase receptors. J. Cell Sci..

[43] Gumina V., Onesto E., Colombrita C., Maraschi A., Silani V., Ratti A. (2019). Inter-Species Differences in Regulation of the Progranulin–Sortilin Axis in TDP-43 Cell Models of Neurodegeneration. Int. J. Mol. Sci..

[44] Consortium U. (2019). UniProt: A worldwide hub of protein knowledge. Nucleic Acids Res..

[45] Humphrey W., Dalke A., Schulten K. (1996). VMD: Visual molecular dynamics. J. Mol. Graph..

[46] Vajda S., Yueh C., Beglov D., Bohnuud T., Mottarella S.E., Xia B., Hall D.R., Kozakov D. (2017). New Additions to the ClusPro Server Motivated by CAPRI. Proteins.

